# The Modulation of Error Processing in the Medial Frontal Cortex by Transcranial Direct Current Stimulation

**DOI:** 10.1155/2013/187692

**Published:** 2013-04-17

**Authors:** Lisa Bellaïche, Manish Asthana, Ann-Christine Ehlis, Thomas Polak, Martin J. Herrmann

**Affiliations:** ^1^Department of Psychiatry, Psychosomatics and Psychotherapy, University Hospital Würzburg, Füchsleinstra*β*e 15, 97080 Würzburg, Germany; ^2^Department of Psychiatry and Psychotherapy, University Hospital Tübingen, Calwerstr. 14, 72076 Tübingen, Germany

## Abstract

*Background*. In order to prevent future errors, we constantly control our behavior for discrepancies between the expected (i.e., intended) and the real action outcome and continuously adjust our behavior accordingly. Neurophysiological correlates of this action-monitoring process can be studied with event-related potentials (error-related negativity (ERN) and error positivity (Pe)) originating from the medial prefrontal cortex (mPFC). Patients with neuropsychiatric diseases often show performance monitoring dysfunctions potentially caused by pathological changes of cortical excitability; therefore, a modulation of the underlying neuronal activity might be a valuable therapeutic tool. One technique which allows us to explore cortical modulation of neural networks is transcranial direct current stimulation (tDCS). Therefore, we tested the effect of medial-prefrontal tDCS on error-monitoring potentials in 48 healthy subjects randomly assigned to anodal, cathodal, or sham stimulation. *Results*. We found that cathodal stimulation attenuated Pe amplitudes compared to both anodal and sham stimulation, but no effect for the ERN. *Conclusions*. Our results indicate that cathodal tDCS over the mPFC results in an attenuated cortical excitability leading to decreased Pe amplitudes. We therefore conclude that tDCS has a neuromodulatory effect on error-monitoring systems suggesting a future approach to modify the sensitivity of corresponding neural networks in patients with action-monitoring deficits.

## 1. Introduction

Performance monitoring is a major executive function, which allows for an online adaptation of behavior according to internal goals and standards and includes the process of error monitoring [1]. In order to accomplish goal-oriented behavior and prevent performance errors, we constantly control the outcome of our actions in order to detect a discrepancy between the expected (i.e., intended) and the real action-outcome, and continuously adjust our behavior accordingly. On the neurophysiological level, erroneous actions are accompanied by a frontocentral negativity (termed error negativity (Ne) or error-related negativity (ERN)) and a corresponding centroparietal positivity (error-positivity, Pe [2]). The ERN typically occurs within the first 100 ms after an erroneous response, while the Pe occurs within 200–450 ms after an incorrect response. Previous studies have shown that the medial frontal cortex (MFC) plays a key role in action monitoring [3]. The premotor/supplementary motor area (Brodman area 6) and caudal anterior cingulate cortex (ACC) have been identified as main generators of the ERN, whereas source localization studies with LORETA (low resolution electromagnetic tomography) localized the generator of the Pe within Brodmann area 24 of the ACC [4]. These results suggest that the ERN and Pe stand for distinct components of error monitoring. While the ERN is thought to be an early indicator of errors that is not dependent on conscious error detection, the Pe component is more likely to reflect conscious aspects of error monitoring depending on error awareness [5]. Brain-injured patients and patients with psychiatric disorders have been found to show performance-monitoring deficits with a corresponding modulation of the electrophysiological correlates of error monitoring [6–9]. All these examples of performance monitoring dysfunctions caused by pathological changes of cortical excitability suggest that a modulation of medial frontal brain areas that are relevant for error processing and performance monitoring might be a valuable additional tool in the therapy of psychiatric disorders.

Noninvasive neurostimulation techniques such as tDCS permit to induce neuroplasticity in human brains and thus allow us to explore cortical modulation of neural networks. tDCS is a non-invasive, pain-free procedure that is able to modulate cerebral excitability by applying weak currents of ~1 mA through surface electrodes to the scalp. The basic principle of the method is that anodal stimulation with positive charging leads to a depolarization of the membrane potential and an increase of cortical excitability, while cathodal stimulation with negative charging leads to hyperpolarization of the resting membrane potential and a decreased firing rate of cortical neurons [10]. Accordingly, both protocols have been shown to result in modified amplitudes of different ERP components [11]. The first approaches that were taken with tDCS aimed at producing localized changes of motor cortex excitability [10]. Soon after these first “proof-of-principle” studies, further studies emerged that explored the modulation of neural networks implicated in human cognitive functions such as working memory [12], verbal fluency [13], or implicit learning [14], were originally designed to address patients with various neurological or psychiatric disorders, and were also conducted with a neuroergonomic mindset.

Based on these findings and considerations, our study aimed at investigating neurophysiological correlates of error monitoring (ERN, Pe) via EEG recordings during stimulation of the brain with weak direct current. Based on previous functional imaging and ERP studies that consistently indicated the ACC (and neighboring regions) as the neuroanatomical source of both the ERN and the Pe, we realized an experimental setup in which the participants underwent DC stimulation over the medial frontal cortex while performing a modified version of the Eriksen flanker task as an experimental paradigm to test response conflict and error monitoring during an EEG recording. According to previous studies investigating motor and cognitive functions, in which a dual-polarity effect for stimulation applied over different cortical areas emerged [15–18], we hypothesize that stimulation with weak direct current over the mPFC might lead to an anodal-excitatory effect under the stimulated area with increased amplitudes of the ERN and Pe, whereas cathodal stimulation might have an inhibitory impact, resulting in smaller amplitudes of the ERN and Pe.

A peculiarity of the analyzed event-related potentials is that source localization studies investigating neural generators contributing to the ERN and Pe revealed different subcomponents of the Pe. van Vean and Carter described an early Pe corresponding to caudal ACC activation and a later Pe with rostral ACC and superior parietal cortex activation [19]. Furthermore, a study investigating to what extent ERP correlates are related to error awareness showed an increased late Pe for aware compared to unaware errors, whereas no dissociation between aware and unaware errors was found for the early Pe [20]. These results replicated a study by Nieuwenhuis and colleagues [5]. Based on these findings indicating potentially different neural generators and functional significance of the late Pe compared to the early Pe, we analyze the Pe with different time windows in our study.

A possible interfering effect between an increased excitability through stimulation over one of the tDCS electrodes and an inhibition effect on the brain area under the opposite electrode shall be taken into account. For example, it would be conceivable that an inhibition of the visual system occurs as a consequence of inhibition as one of our tDCS electrodes is placed between Oz and the inion. Therefore, we analyze the P1 as an early component of the event-related potential evoked within the visual cortex.

With the present study, we intend to shed light upon stimulation effects of tDCS on error monitoring by investigating changes of the ERN and Pe systematically during anodal, cathodal, and sham stimulation.

## 2. Materials and Methods

### 2.1. Participants

Forty-eight healthy subjects (21 males, 27 females; mean age: 23.9 years; SD = 2.5, range 20–30) participated in this experiment. We randomly assigned these subjects to three groups receiving either anodal stimulation (6 males, 11 females; mean age: 24.2 years, SD = 2.7), cathodal stimulation (8 males, 7 females; mean age: 24.0 years, SD = 2.1), or sham stimulation (7 males, 9 females; mean age: 23.5 years, SD = 2.5). All participants received a verbal and written explanation of the purpose of the experiment and gave their written informed consent to participate in the study. All procedures involved were in accordance with the Declaration of Helsinki. The study was approved by the Ethical Review Board of the Medical Faculty of the University of Wuerzburg. All subjects were right handed according to the Edinburgh Handedness Inventory. Exclusion criteria were self-reports of current or former neurological or psychiatric disorders, current or former use of medication affecting the central nervous system, pregnancy or a history of intracranial metal implantation, cochlea implant, or cardiac pacemaker. Information about these criteria was obtained by questionnaires. All subjects underwent neuropsychological tests including the Center for Epidemiological Studies Depression Scale (CED-S) in its German version (ADS-K, Allgemeine Depressionsskala) in its short version [21], the Anxiety Sensitivity Index [22] (ASI-3), and the Adult Self Report Scale [23] (ASRS) as a screening for attention deficit hyperactivity disorder (ADHD). In addition to that, mood changes were monitored using the Positive and Negative Affect Schedule (PANAS [24]) before and after stimulating with tDCS.

### 2.2. Procedure

During the experiment, participants were sitting in a darkened and sound-attenuated cabin in front of a computer screen with EEG- and tDCS-electrode montage on the surface of their head as described below. First, they were asked to close their eyes and relax while a 3-minute resting EEG was recorded. Following a short practice session of a modified version of the Eriksen flanker task, we then started our anodal, cathodal, or sham DC stimulation with a total duration of 22 minutes. After a period of 2 minutes of forerun to maximize the effect of the stimulation, we instructed the participants to perform the Eriksen flanker task during a period of 20 minutes while simultaneously recording the event-related potentials until the end of the DC stimulation. We thus used a so-called online approach, in which tDCS and EEG-recording overlapped in time, so that we could test how cortical stimulation instantaneously modulated the activity of the stimulated brain area. The experiment ended with another 3 minutes of resting EEG.

### 2.3. tDCS

For stimulation we used a DC stimulator by Neuroconn, Ilmenau (Germany) approved for use in humans. During each tDCS trial, participants received either anodal, cathodal, or sham stimulation which is indistinguishable for the subjects (as described below).

A pair of rectangular surface electrodes (5 cm × 7 cm; 35 cm^2^) were coated with Ten20 Conductive Paste (Weaver and Company, Aurora CO, USA) and then applied to the participants scalp (Current density: 0.0286 mA/cm^2^). Following the literature, we positioned the tDCS electrodes according to the international 10–20 system. As we intended to stimulate the medial prefrontal cortex (mPFC), we decided to place one electrode horizontally over Fpz. As previous investigations have shown that maximizing the distance between both electrodes results in a decrease of current shunted through the head and an increase of current density in depth, we placed our second electrode between the inion and Oz, also in a horizontal direction [25]. Placement of the electrodes was identical for sham and real stimulation. For anodal versus cathodal stimulation, the polarity of the frontal electrode was switched.

At the beginning of the stimulation period, the current was ramped up over the first 10 seconds. After that, a constant current of 1 mA was applied for a period of 22 minutes. Current delivery of up to 2 mA has been shown to be safe and painless for healthy volunteers [26]. The impedance was controlled by the stimulator and was below 20 kΩ at all times. After 22 minutes of constant current, current was ramped out over another 10 seconds. For sham stimulation, the electrode placement, fade-in time, and current intensity were identical, but the stimulation was aborted/terminated after 2 minutes, that is, at the beginning of the EEG recording and performance of the Eriksen flanker task. By starting sham stimulation exactly like a real stimulation, with an identical mild tingling that is felt on the skin under the electrode in the first seconds of real stimulation, sham stimulation could not be distinguished from real stimulation by our subjects.

### 2.4. EEG Recording

The EEG was recorded with an AC amplifier (MRAmp, Brain Products GmbH, Munich, Germany) and an ActiCap electrode system (Brain products GmbH, Munich, Germany) with 32 electrodes. The electrodes were placed according to the international 10–20 system with F7, F3, Fz, F4, F8, FC5, FC1, FC2, FC6, T7, C3, Cz, C4, T8, TP9, CP1, CP2, TP10, P7, P3, Pz, P4, P8, P09, O1, Oz, O2, and PO10. Another two electrodes were placed above and below the right eye. Ground electrode was placed at CP5. All electrodes were referenced to CP6 and rereferenced offline to an average reference. All impedances of the electrodes were below 10 kΩ and always below 5 kΩ for the reference and ground electrode. EEG was recorded with a sampling rate of 1000 Hz and a bandpass filter of 0.1–100 Hz as well as a notch filter of 50 Hz. During the resting state EEG recordings at the beginning and at the end of the experiment, no tDCS was applied.

### 2.5. Task

In our experiment, we used a modified version of the Eriksen flanker task [27], an experimental paradigm eliciting response conflict which is often used to study error-monitoring potentials. The participants sat in front of a monitor with a black background, where four different combinations of 5 arrow heads were presented (<<<<<, >>>>>, >><>>, <<><<) for 125 ms each. The participants were instructed to focus on the center arrow head while ignoring the flanking “distracters,” and to press a response button matching the direction of the central arrow with their right or left index finger as quickly and accurately as possible. 750 ms after their response a feedback signal indicated if their response was correct or not. A plus sign appeared for 500 ms if the answer was correct, a minus sign indicated an incorrect or missing response, and an exclamation mark indicated that the response was not given in time. Prior to the beginning of the EEG recording, a timeframe for a correct and timely response was established by a practice session consisting of 48 trials during which the median reaction time for each participant was determined. In our experiment, the modified Eriksen flanker task contained 280 trials presented in one continuous block. In the ongoing EEG, the presentation of the stimuli, the motor responses, and the feedback signals were tagged by different markers. Participants underwent DC real or sham stimulation during the entire performance of the flanker task.

To analyze the behavioral effects, we calculated the post-error slowing (PES), defined as reaction times in correct trials after a correct trial minus the reaction times in correct trial after an incorrect trial.

### 2.6. Analysis of EEG Data

The EEG data was analyzed with the BrainVision Analyzer Version 2.0 (Brain Products, Munich, Germany). In a first step, the ocular electrodes were linked to one bipolar channel monitoring vertical eye movements. In a second step, eye blinks were corrected by applying an ocular correction algorithm [28]. Data was rereferenced to an average reference and was then segmented into EEG epochs following correct or incorrect responses starting −100 ms before and ending 600 ms after the subject's key press. Based upon these EEG epochs, average ERP waves were calculated for each participant after correct and incorrect responses. Before that, EEG epochs with amplitudes exceeding ±100 *μ*V or voltage steps of more than 100 *μ*V/sampling point were excluded by an automatic artefact rejection. Finally, a baseline correction from −100–0 ms was applied. Subjects with anodal stimulation had 45.2 ± 24.5 artefact-free epochs after incorrect responses and 140.9 ± 32.3 artefact-free epochs after correct responses. Participants with cathodal stimulation had 39.9 ± 25.6 artefact-free epochs after incorrect responses and 105.3 ± 44.4 artefact-free epochs after correct responses, while subjects with sham stimulation had 41.9 ± 23.2 artefact-free epochs after incorrect responses and 139.4 ± 46.9 artefact-free epochs after correct responses. Groups did not differ significantly regarding the mean number of artefact-free epochs after incorrect responses (*F*[2,45] = 0.19, *P* = 0.83), but after correct responses (*F*[2,45] = 3.49, *P* < 0.05). This does not have a big influence on our findings, as we were mainly interested in epochs after incorrect responses.

Based on a visual inspection of the grand average waveforms, we individually calculated the mean amplitude for the time frames between 0 and 60 ms after correct and erroneous responses for the ERN and the segments between 100 and 200 ms and between 200 and 300 ms after correct and incorrect responses for the Pe. For both potentials, analyses were conducted for the central electrode position (Cz), as the topographical maps (see Figure 3) showed a brain electrical field distribution that was centred around Cz for both the ERN and the Pe.

The P1 responses as an indicator for an altered processing of visual information were defined as the maximum peak between 80 ms and 150 ms over three occipital electrodes (O1, O2, and Oz according to the international 10/20 system for electrode placement).

### 2.7. Statistical Analysis

For statistical analysis, we used IBM SPSS Statistics, version 19. For testing stimulation effects, we calculated an ANOVA separately for the behavioural data, ERN and Pe with the factor condition (correct or erroneous responses) and tDCS group (cathodal, anodal, sham). In order to test the reaction time data for the phenomenon of “posterror slowing,” the factor “condition” comprised trials following correct responses and trials following incorrect responses for this dependent variable. Greenhouse-Geisser correction was applied if necessary. Univariate ANOVAs were calculated for further analyses of main effects and interactions with planned comparisons comparing the two stimulation conditions with the sham condition using the Fisher's least significant difference (LSD).

## 3. Results and Discussion

### 3.1. Behavioural Data

For the reaction time, a main effect of condition was found (*F*[1,44] = 40.21, *P* < 0.001), with a significant increase of reaction times on trials following erroneous responses (464.5 ± 60.6 ms) compared to trials following correct button presses (440.5 ± 50.8 ms) across stimulation groups (posterror slowing). We did not observe a significant difference between stimulation groups (*F*[2,44] = 2.04, *P* = 0.14) and no significant interaction between the factors “group” and “condition” (*F*[2,44] = 0.041, *P* = 0.96). With respect to the number of incorrect responses, no relevant difference between the three groups emerged (*F*[2,44] = 1.53, *P* = 0.23) with a similar number of incorrect responses in participants of the anodal (41.4 ± 18.9), cathodal (56.7 ± 24.9) and sham group (46.6 ± 30.5).

Posterror slowing (PES) was also directly analyzed as an indicator for a possible stimulation-induced behavioral effect. PES was not significantly influenced by the stimulation protocol (*F*[2,44] = 0.041, *P* = 0.96; see also nonsignificant interaction effect above); specifically, PES was not significantly attenuated after cathodal stimulation (*m* = 23.07 ± 26.30 ms) compared to anodal (*m* = 25.41 ± 29.13 ms) or sham stimulation (*m* = 23.27 ± 20.78 ms).

### 3.2. EEG Data

**Figure 1:**
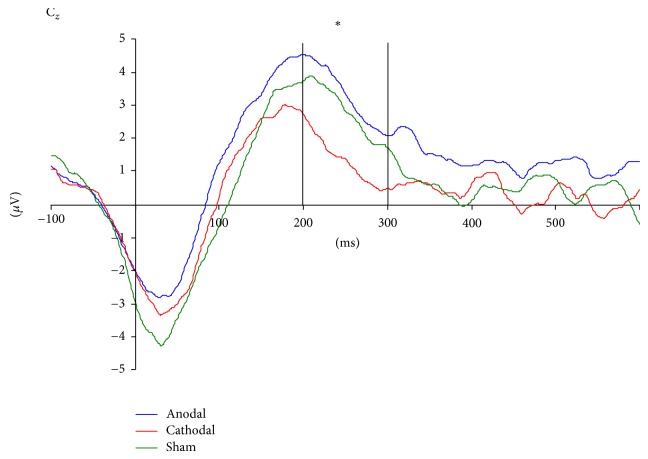
Difference curve (incorrect minus correct responses) over Cz for anodal, cathodal, and sham stimulation; ∗*P* < 0.05.

**Figure 2:**
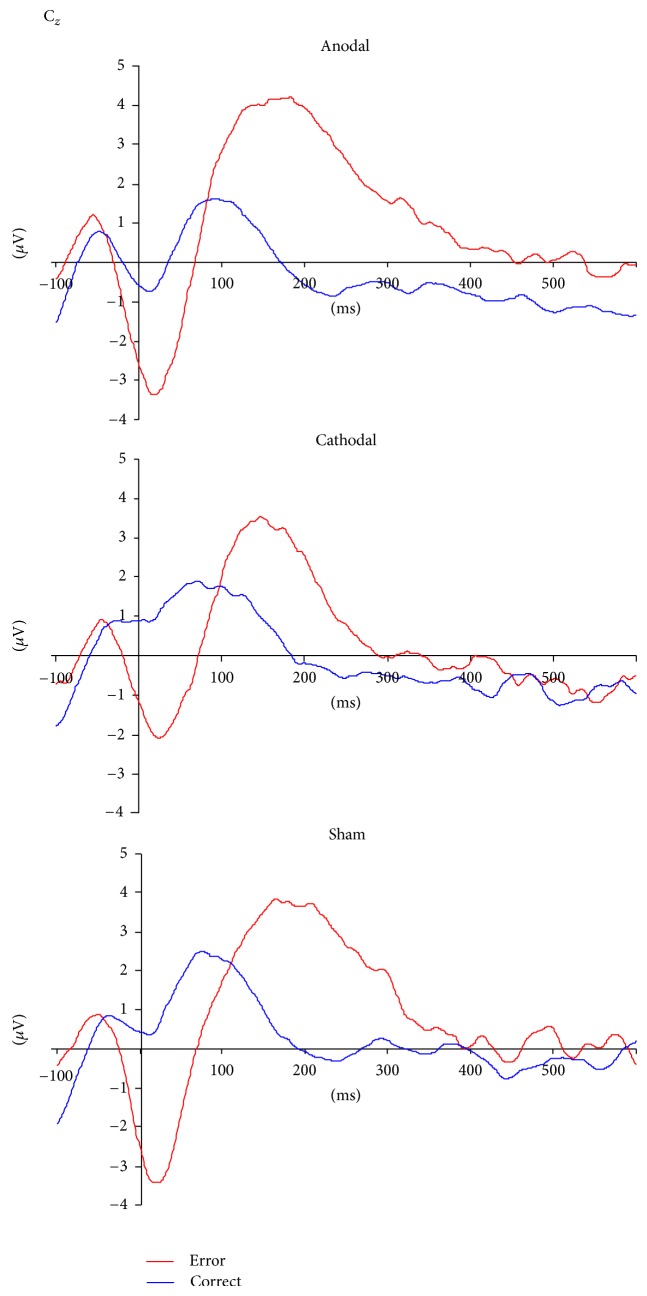
Time curves for incorrect and correct responses over Cz for anodal, cathodal, and sham stimulation; ∗*P* < 0.05.

For the ERN (see Figures 1 and 2), we calculated the mean amplitude over Cz in the time segment of 0–60 ms after correct (*m* = 0.81 ± 2.37 *μ*V) and erroneous (*m* = −2.32 ± 2.93 *μ*V) responses revealing a significant increase of the amplitude after errors compared to correct answers (*F*[1,45] = 66.8, *P* = 0.00001). No main effect of group (*F*[2,45] = 0.91, *P* = 0.41) and no significant interaction effect of stimulation protocol and condition occurred (*F*[2,45] = 0.85, *P* = 0.43).

**Figure 3:**
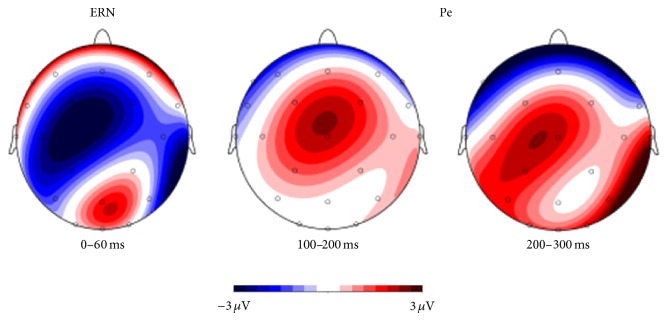
Mean amplitudes for the difference between incorrect and correct responses in the time windows of the ERN (0–60 ms), the first part of the Pe (100–200) and the second part of the Pe (200–300), averaged over all subjects of the three stimulation groups.

For the Pe (see Figure 1), we analyzed two time segments between 100 and 300 ms. With regard to the first time segment between 100 and 200 ms, we found a main effect condition (*F*[1,45] = 53.10, *P* = 0.00001), indicating that mean Pe amplitudes were higher for incorrect (*m* = 3.42 *μ*V ± 2.85) compared to correct (*m* = 0.84 *μ*V ± 2.48) responses; no main effect for tDCS groups (*F*[2,45] = 0.061, *P* = 0.94) and no interaction effect (*F*[2,45] = 1.13, *P* = 0.33) were found. For the second time segment comprising the period between 200 and 300 ms, a main effect for condition was again found (*F*[1,45] = 56.05, *P* = 0.0001), but no main effect for the type of stimulation could be observed (*F*[2,45] = 1.19, *P* = 0.31). However, a significant interaction effect between tDCS group and condition additionally occurred (*F*[2,45] = 3.23, *P* = 0.049). The time curves for both conditions are displayed in Figure 2. The topographical distributions of the components are displayed in Figure 3.

**Figure 4:**
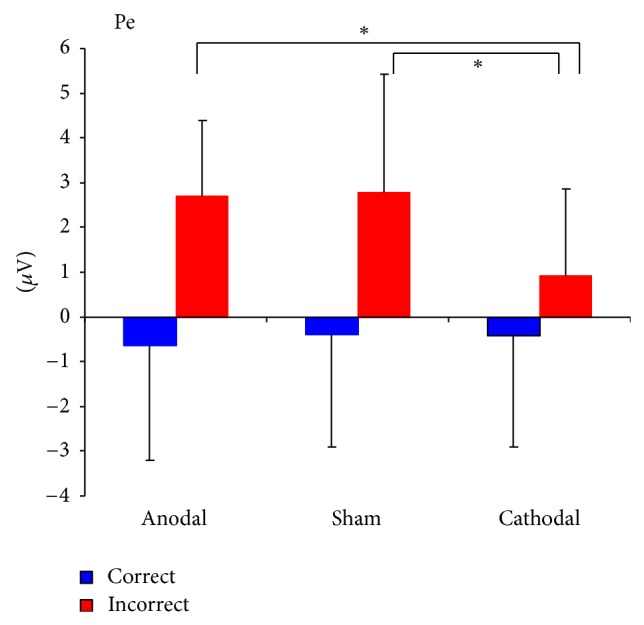
Interaction effect between group and condition: mean amplitude over Cz in the time segment 200–300 ms after correct and incorrect responses under anodal, cathodal, and sham stimulation; error bars indicate standard deviations of the mean. ∗*P* < 0.05.

For further examination of the interaction effect (see Figure 4) between the condition (correct or incorrect response) and the different stimulation groups, we used post hoc ANOVAs to compare group effects for erroneous responses (*F*[2,45] = 3.76, *P* = 0.03) and correct responses (*F*[2,45] = 0.21, *P* = 0.81) separately and found a main effect for the erroneous condition only.

Planned post hoc comparison (LSD) for the erroneous condition showed no significant difference between anodal and sham stimulation (*P* = 0.92). However, comparing cathodal and sham stimulation, we found a significant difference (*P* = 0.019) with reduced Pe amplitudes following cathodal stimulation, even after correction for multiple testing.

Analyzing the latency parameters (in the corresponding time window for the ERN and for a time window including both time frames for the Pe) for the ERN, a significantly shorter latency after correct answers (*m* = 14.33 ± 16.67 ms) compared to incorrect answers (*m* = 21.48 ± 15.76 ms) was found (*F*[1,45] = 8.34, *P* = 0.006). No significant main effect for the type of stimulation (*F*[2,45] = 0.18, *P* = 0.84) and no significant interaction effect (*F*[2,45] = 0.40, *P* = 0.67) were observed. The latency parameters for the Pe revealed no main effect for the different types of stimulation (*F*[2,45] = 1.16, *P* = 0.32) with similar latencies in the anodal (*m* = 168.73 ± 10.84 ms), cathodal (144.63 ± 11.54 ms), and sham group (*m* = 158.12 ± 11.18 ms). Latency of the Pe after a correct reaction (*m* = 146.48 ± 60.24 ms) was significantly shorter than that after an incorrect reaction (*m* = 168.88 ± 45.03 ms) with *F*[1,45] = 7.46 and *P* = 0.01. No interaction effect between condition and type of stimulation emerged (*F*[1,45] = 1.07, *P* = 0.35).

To check whether our tDCS electrode placement leads to a deficient visual processing, which might cause the effect on the Pe, we calculated an analysis of variance (ANOVA) for the P1 amplitudes which did not reveal a main effect “group” over O1 (*F*[2,45] = 0.38, *P* = 0.68), O2 (*F*[2,45] = 0.61, *P* = 0.54), or Oz (*F*[2,45] = 0.46, *P* = 0.63).

## 4. Discussion

The main purpose of our study was to shed light upon stimulation effects on error monitoring by applying tDCS during an Eriksen flanker task and investigating the changes of the ERN and Pe systematically after anodal, cathodal, or sham stimulation. As expected [2], a main effect “condition” was observed for the ERN and Pe; that is, the amplitudes of the ERN and Pe were significantly higher after incorrect responses than that after correct responses. For tDCS effects, we found that cathodal stimulation over the medial prefrontal cortex attenuated subcomponents of Pe amplitudes compared to both anodal stimulation and sham stimulation, but no effect for the ERN. We therefore conclude that tDCS shows an inhibition effect of cortical excitability during cathodal stimulation, while no enhancement of excitation by anodal stimulation could be observed. Originally, the assumption that anodal stimulation increases cortical excitability whereas cathodal stimulation causes the opposite was based upon studies applying direct current stimulation over the human motor cortex [10, 29, 30]. However, the dual effect of anodal excitation and cathodal inhibition has not been reproduced in all subsequent studies testing effects of tDCS over nonmotor regions on cognitive functions. Some studies describe an anodal excitation effect only [12], while others observed only a cathodal inhibition effect as we did [25]. A recent meta-analysis reviewing publications of motor studies and cognitive studies with tDCS found a lot of heterogeneity particularly in cognitive studies and concluded that the probability to achieve the anodal-excitability and cathodal-inhibition effect in a cognitive study is only about 0.16 [31]. As a possible reason for this heterogeneity, the authors argue that cognitive tasks involve various cognitive regions of the brain. Therefore, when modulating one part of this brain network, it is more difficult to induce any change in corresponding outcome measures than it would be for motor effects (e.g., concerning MEPs) which typically involve only the stimulated motor cortex. Beyond that, the fact that a process as complex as the performance of a cognitive task, that involves various interactions between different brain regions, is so highly vulnerable to every external noise, making it extremely difficult to investigate the specific effects of tDCS and might be one reason for the finding of a cathodal inhibition effect only.

Another potential explanation is that in a “normal” mPFC (and we act on the assumption that our healthy participants all have a well functioning mPFC), a tDCS-induced impairment is possible due to cathodal inhibition, but that the same principle (with an opposite outcome) cannot automatically be applied for anodal stimulation. It is possible that the function of a “healthy” mPFC with regard to error processing cannot be enhanced by anodal stimulation due to a ceiling effect [25]. In any case, we would like to summarize that, in the present study, the effect of tDCS stimulation was specific for cathodal stimulation and not a nonspecific effect of direct current application.

Another central issue of our study is the question of whether or not the brain region we targeted has actually been reached. As stated above, the surface of the cerebral cortex is folded, so that a large part of the cortex is located deep within the sulci where neurons are differently oriented. In early animal experiments, surface-anodal tDCS enhanced activity and surface-cathodal tDCS reduced activity of superficial cortical neurons, whereas neurons situated deep in the cortical sulci (and thus differently oriented) were oppositely affected [32]. Therefore, it is essential to take into account that not only electrode placement itself is an important parameter determining the electrical stimulation effects on the ACC, but that the induced currents in the brain depend on tissue characteristics, which might even skew stimulation effects. One might ask in this context if current density was high enough to modify excitability. Different calculations of current density distribution based on a spherical model of the head can help us to assess the current density during tDCS [33]. Calculations suggest that the average current flows in the expected direction independently of gyri and sulci of the brain and that an increasing distance between electrodes increases the current density in depth. Furthermore, a minimum of 0.017 mA/cm^2^ has been described to be necessary to modify excitability by Nitsche and Paulus [10]. The applied current density in our study was 0.029 mA/cm^2^ and the reference electrode was placed at a maximum distance from the first electrode over Fpz, so that these parameters are in accordance with previous tDCS studies demonstrating a relevant effect of DC stimulation. Nevertheless, computer-based head models would be useful to further analyze current distribution effects [34].

Another method that could be helpful to provide evidence for the influence of the stimulation on brain activity is electroencephalographic power spectrum analysis which has been used in recent studies to demonstrate that tDCS modulates resting state EEG parameters [35–37]. In these combined EEG and tDCS studies, the power of different frequency bands was measured, indicating a direct impact of tDCS on oscillatory activity of the brain. Keeser and colleagues [36] stimulated the left prefrontal cortex by anodal tDCS and observed decreased delta activity over the left frontopolar cortex and enhanced beta activity over the right frontocentral cortex. LORETA source localization revealed that the reduction of the delta power could be explained by a reduced activation in Brodman area 25 and 32 (subgenual prefrontal and anterior cingulate cortex). These results confirm that cortical tDCS can indeed induce a modulation of deeper brain structures such as the ACC, a key structure for error monitoring.

With regard to our electrode placement, another aspect that has to be taken into consideration is an inhibition of the visual system as a result of a decreased cortical excitability under the electrode placed between Oz and the inion. This deficient visual processing might lead to a modification of visual information that could be relevant for our behavior. Therefore, we analysed the P1 as an early component of event-related potentials which are generated in the visual cortex and found no relevant difference for the amplitudes of the P1 in the three stimulation groups. We therefore assume that DC stimulation under the occipital electrode had no relevant effect on our experimental setting. The distance between the occipital electrode and Oz seems to be large enough to avoid an undesirable modulation of the visual system

Another point that needs further consideration is the fact that no significant behavioral effects of tDCS were found in our study. Generally speaking, goal-directed behavior includes the monitoring of ongoing actions on the one hand and adjustment of behavior on the other hand, both of which need to be linked at some point to organize behavior. Neuroanatomically, there is evidence for a functional interaction between the mPFC and the lateral prefrontal cortex (LPFC), with prominent models of cognitive control assuming that the mPFC signals error detection to the LPFC where regulatory processes lead to adjustment of behavior [38]. Regarding posterror behavior, Danielmeier and Ullsperger [39] describe that there are at least three types of post-error behavioral adjustment, that is, post-error-slowing (PES), post-error reduction of interference (PERI), and post-error improvement of accuracy (PIA), which partly take place in parallel. Regarding PES, which was measured in our study, they assume that PES can be related to cognitive control processes and to inhibitory motor processes or reflect attentional reorientation. The expectation that PES should be attenuated by an inhibitory effect on the mPFC addresses the cognitive control theory according to which post-error adjustments are triggered by top-down signals. Studies investigating correlations between PES and mPFC activity, however, show inconsistent results. While some studies could show a correlation between PES and mPFC activation [40], other studies describe contradictory findings [3]. As one possible reason for these inconsistent findings, it has been discussed that PES and mPFC are only linked indirectly via increased activity in the motor system [41]. Taken together, it should be noted that, when investigating posterror behavioral adjustments, the problem is that many adjustments are executed simultaneously. Hence, measuring only mean reaction times—as we did in our experiment—is not always sufficient to reveal discrete behavioral changes, as it conceals temporal dynamics of error-monitoring processes. It can, for example, be difficult to distinguish the monitoring signal indicating the need for control from subsequent control implementation. To summarize, we restricted our behavioral analyses to effects on mean reaction times, thus providing an only incomplete picture of post-error behavioral adjustments by means of PES. Therefore, relevant behavioral effects by tDCS interventions in our study were not necessarily expected and it cannot be concluded that no post-error control implementation (or modulation thereof) has taken place.

Another argument which illustrates that behavioral data are sometimes not as sensitive as neural correlates comes from a preclinical study. In this study, we used the same flanker task to investigate whether error processing is deficient in students with high levels of ADHD symptoms [42]. As hypothesized, we found decreased Pe amplitudes with an increased number of symptoms of inattention, but without an effect of ADHD symptoms on the behavioral level of post-error slowing.

In summary, the present study aimed at modulating medial frontal cortical areas relevant for error detection and action monitoring via tDCS. Our results indicate that cathodal tDCS applied over the mPFC results in an attenuated cortical excitability reflected in a decreased amplitude of subcomponents of the Pe. At this point, we could show an effect of single-session tDCS on the electrophysiological correlates of error monitoring in healthy subjects, even if it is rather small. Nevertheless, further research is required to unravel whether the effect of the tDCS on the late Pe is of functional relevance. Based upon these investigations, transcranial direct current stimulation could become a future approach to modify the sensitivity of corresponding neural networks. Future studies should be conducted to shed further light on the (patho)physiology of the underlying performance monitoring systems on cellular and system levels to allow for an optimization of stimulation-induced tDCS effects and to establish tDCS as a valuable therapeutic option for patients with error-monitoring dysfunctions.
